# DNA methylation of the *Fthl17 *5’-upstream region regulates differential *Fthl17 *expression in lung cancer cells and germline stem cells

**DOI:** 10.1371/journal.pone.0172219

**Published:** 2017-02-16

**Authors:** Nana Aoki, Kentaro Mochizuki, Yasuhisa Matsui

**Affiliations:** 1 Cell Resource Center for Biomedical Research, Institute of Development, Aging and Cancer (IDAC), Tohoku University, Sendai, Miyagi, Japan; 2 Department of Developmental Biology and Neurosciences, Graduate School of Life Sciences, Tohoku University, Sendai, Miyagi, Japan; 3 The Japan Agency for Medical Research and Development-Core Research for Evolutional Science and Technology (AMED-CREST), Tokyo, Japan; 4 Center for Regulatory Epigenome and Diseases, Tohoku University School of Medicine, Sendai, Miyagi, Japan; Universidad de Chile, CHILE

## Abstract

The *Ferritin heavy polypeptide-like 17* (*Fthl17*) gene is a member of the cancer/testis antigen gene family, and is preferentially expressed in cancer cells and in testis. Although DNA methylation has been linked to the regulation of human *FTHL17* gene expression, detailed epigenetic regulation of its expression has not been investigated. To address this, we assessed the epigenetic regulation of murine *Fthl17* gene expression in cancer cells and germ cells. *Fthl17* was more highly expressed in testis, a murine lung cancer cell line, KLN205, and in germline stem cells (GSCs) than in normal lung tissues. Furthermore, the *Fthl17* expression level in GSCs was significantly higher than in KLN205 cells. We performed bisulfite-sequencing and luciferase (luc) reporter assays to examine the role of DNA methylation of the *Fthl17* promoter in the regulation of *Fthl17* expression. In KLN205 cells, testis, and GSCs, the *Fthl17* 5’-upstream region was hypo-methylated compared with normal lung tissues. Luc reporter assays indicated that hypo-methylation of the -0.6 kb to 0 kb region upstream from the transcription start site (TSS) was involved in the up-regulation of *Fthl17* expression in KLN205 cells and GSCs. Because the -0.6 kb to -0.3 kb or the -0.3 kb to 0 kb region were relatively more hypo-methylated in KLN205 cells and in GSCs, respectively, compared with other regions between -0.6 kb to 0 kb, those regions may contribute to *Fthl17* up-regulation in each cell type. Following treatment with 5-Azacytidine, the -0.3 kb to 0 kb region became hypo-methylated, and *Fthl17* expression was up-regulated in KLN205 cells to a level comparable to that in GSCs. Together, the results suggest that hypo-methylation of different but adjacent regions immediately upstream of the *Fthl17* gene contribute to differential expression levels in lung cancer cells and GSCs, and hypo-methylation of the TSS-proximal region may be critical for high level expression.

## Introduction

Cancer/testis antigen (CTA) genes were identified as human tumor-specific antigen genes which are specifically expressed in normal testis and various cancers, though their functions in germ cells and tumors are still unclear. CTA genes are classified as X-linked or autosomal [1]. In the testis, CTA genes on the X-chromosome and on autosomes are preferentially expressed in spermatogonia and spermatocytes, respectively [1]. Furthermore, X-linked CTA genes are often expressed in melanoma, bladder, lung and ovarian cancers, and in hepatocellular carcinoma.

DNA methylation generally represses gene expression. Werber *et al*. first reported the correlation between DNA methylation and the expression of CTA genes, and found that the CTA gene, *MAGE-A1*, was activated by treatment of a human melanoma cell line with the DNA demethylating agent, 5-aza-2’-deoxycytidine (5-aza-CdR) [2]. In addition, the expression of *MAGE-A1*, and other *MAGE-A* family genes, correlated with the DNA methylation levels of their respective promoter regions in human tumor tissues and in cancer cell lines [3,4]. In mice, many CTA gene homologs are transcribed from hypo-methylated regions in primordial germ cells [5], implicating DNA demethylation in the regulation of their expression. Taken together, these data suggest that DNA methylation plays a fundamentally important role in regulating the expression of CTA genes.

Histone H3 lysine 9 (H3K9) dimethylation (H3K9me2) is a repressive epigenetic modification that is involved in the regulation of CTA gene expression [6,7]. Shinkai *et al*. found that H3K9me2 levels at *Mage-a* promoters were reduced, and the expression of Mage-a family members was up-regulated in knockout mouse embryonic stem cells of G9a and/or GLP, both of which are H3K9-specific methyltransferases. However, the roles of G9a/GLP and H3K9me2 in the regulation of expression of other CTA genes in cancer cells remain unclear. In human colon cancer cells, the expression of the CTA genes, *MAGE-A1*, *NY-ESO-1*, *XAGE-1*, remained unchanged in response to G9a and/or GLP knockdown [8]. However, when the G9a knockdown cells or control cells were treated with 5-aza-CdR, the expression of these CTA genes in the knockdown cells was dramatically up-regulated compared to control cells. These results suggest that DNA methylation and histone modifications cooperatively regulate CTA gene expression, and the relative importance of the two mechanisms varies among CTA genes and cell types.

The human CTA gene, *Ferritin heavy polypeptide-like 17* (*FTHL17*), is expressed in bladder, breast and lung carcinomas and in testis, but not in other normal somatic tissues [9]. Although *FTHL17* encodes a ferritin-like protein, it has neither ferroxidase activity nor a role in iron storage [10], and its cellular roles remain enigmatic. *FTHL17* is not expressed in human melanoma, but similar to *MAGE-A1*, it is up-regulated in response to treatment with a DNA demethylating agent [9], implicating DNA methylation in the regulation of its expression. The mouse ortholog of *FTHL17* is a paternally imprinted gene on the X-chromosome [11], which is highly expressed in germline stem cells (GSCs), and minimally expressed in brain, ovarian cumulus and liver [5]. Although, imprinted genes are generally regulated by DNA methylation, differentially methylated regions were not found within or adjacent to the *Fthl17* gene in male and female blastocysts [11]. Thus, a role for methylation-associated regulatory regions in the control of *Fthl17* expression has not yet been established.

Although DNA methylation is involved in the regulation of CTA gene expression as described above, little is known about the similarities and differences in regulatory mechanisms that control CTA gene expression in cancer cells and germ cells. In this study, we addressed the role of epigenetic regulation, and DNA methylation in particular, in the regulation of *Fthl17* gene expression in cancer cells and germ cells.

## Materials and methods

### Cell culture

KLN205 mouse lung cancer cell line was obtained from Cell Resource Center for Biomedical Research (Tohoku University, Japan). KLN205 cells were cultured in Minimum Essential Medium Eagle (MEM) (Sigma-Aldrich) containing 10% fetal bovine serum (FBS) (Biosera) and MEM Non-Essential Amino Acids (NEAA) (Gibco) at 37°C in 5% CO2. Germline stem cells (GSCs) were kindly gifted by Dr. T. Shinohara (Kyoto University, Japan) and were cultured as described previously [12]. Briefly, culture medium for GSCs was StemPro-34 SFM (Gibco) supplemented with StemPro supplement (Gibco), 25 μg/ml insulin (Sigma-Aldrich), 100 μg/ml transferrin (Sigma-Aldrich), 60 μM putrescine (Sigma-Aldrich), 30 nM sodium selenite (Sigma-Aldrich), 6 mg/ml D-(+)-glucose (Gibco), 30 μg/ml pyruvic acid (Gibco), 1 μl/ml DL-lactic acid (Sigma-Aldrich), 5 mg/ml bovine albumin (Sigma-Aldrich), 2 mM L-glutamine (Gibco), 5×10^−5^ M 2-mercaptoethanol (Sigma-Aldrich), MEM vitamin solution (Gibco), MEM NEAA solution (Gibco), 10^−4^ M ascorbic acid (Sigma-Aldrich), 10 μg/ml d-biotin (Sigma-Aldrich), 30 ng/ml b-estradiol (Sigma-Aldrich), 60 ng/ml progesterone (Sigma-Aldrich), 20 ng/ml mouse epidermal growth factor (Sigma-Aldrich), 10 ng/ml human basic fibroblast growth factor (Sigma-Aldrich), 10^3^ U/ml leukemia inhibitory factor (millipore), 10 ng/ml recombinant rat glial cell line-derived neurotrophic factor (R&D Systems) and 1% FBS (Morgate). The cells were maintained on a feeder layer of mitotically inactivated mouse embryonic fibroblast.

### Collection of tissue

C57BL/6J mice were purchased from Japan SLC. The mice were kept and bred in the Animal Unit of the Institute of Development, Aging and Cancer (Tohoku University), an environmentally controlled and specific pathogen-free facility, according to the guidelines for experimental animals defined by the facility. Animal protocols were reviewed and approved by the Tohoku University Animal Studies Committee. The mice were killed by cervical dislocation to collect tissues. Normal mouse lung and testis tissues were surgically collected from three male mice at 12 months, 6 months and 8 weeks of age. They were then washed in Dulbecco’s phosphate buffered saline (DPBS) (Gibco) and immediately frozen in liquid nitrogen and stored -80°C.

### Real-time PCR

Total RNA was extracted from tissues or cells using RNeasy Midi Kit (QIAGEN) or RNeasy Plus Mini Kit (QIAGEN) according to the manufacture’s instruction. RNAs were reverse-transcribed using SuperScript III reverse transcriptase (Invitrogen) and random primers (Promega). Real-time PCR was performed using Power SYBR Green PCR Master Mix (Applied Biosystems). Thermal conditions were 2 min at 50°C, 10 min at 95°C, and 45 cycles of 15 sec at 95°C and 60 sec at 60°C. Sequences of the primers used for the PCR reaction were shown in Table 1. *Arbp* was used as an internal control. The relative expression was analyzed using comparative CT method. If a Ct value was not estimated in 45 cycles of amplification, the expression level was considered as 0.

**Table 1:** Primers used for quantitative RT-PCR, bisulfite sequencing and cloning.

Gene Symbol (position)		Primer sequence (5’-3’)
Quantitative RT-PCR primers		
*Fthl17*	F	GTTGTACAGCTCAAGTGCCTG
	R	CCAGTCATAGTTCTGCTGCAT
*Arbp*	F	AGATTCGGGATATGCTGTTGGC
	R	TCGGGTCCTAGACCAGTGTTC
Bisulfite sequencing primers		
*Fthl17* (-1.4 kb)	F (outer)	TAGTATTTGGGAGGAAGAGGTAGGT
	R (outer)	AAAAACTCTTCAAAACTCCAACTCC
	F (inner)	TTTAGGTAGTAAAAATAAGAAAGGTGGTG
	R (inner)	AACAACTCTACCCAACAAATAAATATACAA
*Fthl17* (-1.0 kb)	F (outer)	AAGGAGTTGGAGTTTTGAAGAGTTT
	R (outer)	CATCATTTCAACCCCAAAAATTATATA
	F (inner)	GTTTTTTGATATTAAATGGAGATGGAGT
	R (inner)	CATCATTTCAACCCCAAAAATTATATA
*Fthl17* (-0.6 kb)	F (outer)	AAATGATGTTATGGATTGGGTGTTAGTAT
	R (outer)	TAATCTCAACAAAAAAACTTAAAAAAAA
	F (inner)	AAATGATGTTATGGATTGGGTGTTAGTAT
	R (inner)	AAAAAACATCAATAAAAAATAAAATTAAAA
*Fthl17* (-0.3 kb)	F (outer)	TTTATTTTTTTTATTAGTTTTGAGGTTGAA
	R (outer)	AACAAAAAACACAATCCCACAAT
	F (inner)	GGTTTTAATTTTATTTTTTATTGATGTTTT
	R (inner)	AAACACAATCCCACAATCCC
Primers for cloning *Fthl17* promoters		
*Fthl17* (-1.7 kb)	F (+SpeI)	GACTAGTGACCTCAGAACAAGCC
	R (+BamHI)	GGATCCAAGAACACAGTCCC
*Fthl17* (-0.6 kb)	F (+SpeI)	ACTAGTGCACCAACACTCCTATCA
	R (+BamHI)	GGATCCAAGAACACAGTCCC

F: Forward primer sequence; R: Reverse primer sequence. Primer sequences for cloning contain endonuclease restriction site on 5’ end.

### Bisulfite-sequencing analysis

Total DNA was isolated from tissues or cells using DNeasy Blood & Tissue Kit (QIAGEN) according to the manufacture’s instruction. Bisulfite conversion of the DNA was performed using EZ DNA Methylation-Direct Kit (ZYMO Research). Nested PCR was performed using BIOTAQ HS DNA Polymerase (BIOLINE). The sequences of the PCR primer were designed with MethPrimer (http://www.urogene.org/cgi-bin/methprimer/methprimer.cgi) [13]. The primer sequences are shown in Table 1. The PCR products were gel-purified, sub-cloned into the pGEM-T Easy vector (Promega), and sequenced using an ABI PRISM 3100-Avant Genetic Analyzer (Applied Biosystems). Sequence data were analyzed with Quantification tool for Methylation Analysis (http://quma.cdb.riken.jp/top/quma_main_j.html) [14]. The data were obtained from two independent experiments.

### Luciferase reporter assay

The 1.7 kb and 0.6 kb 5’-upstream region of *Fthl17* were amplified and sequenced. Each sequence was cloned into the CpG-free pCpGL-basic Luciferase vector [15] by ligation using TaKaRa DNA Ligation Kit <Mighty Mix> (TaKaRa). Luciferase reporter constructs were either mock-treated or methylated in vitro with SssI CpG methyltransferase for 4 h at 37°C and purified with the QIAquick Purification Kit (QIAGEN). 500 ng of each reporter plasmid and 50 ng of Renilla phRL-TK control vector (Promega) were co-transfected into KLN205 cells and GS cells using Lipofectamine LTX (Invitrogen) and Neon Transfection System (Invitrogen), respectively. After 48h, cells were lysed. Then, the relative Luciferase activities were analyzed using the Dual-Luciferase Reporter Assay System (Promega) on a Lumat LB 9507 (Berthold). Firefly Luciferase activity of individual transfections was normalized against Renilla Luciferase activity.

### 5-Azacytidine treatment

KLN205 cells were treated with 5-Azacytidine (5-Aza). Treated cells were cultured for 72 h with 10 μM 5-Aza. Real-time PCR and bisulfite-sequencing analysis were performed as described above.

### Searching transcription factor binding sites

Transcription factor binding motifs in the -0.3 kb to 0 kb region of the *Fthl17* promoter were analyzed by JASPAR database (http://jaspar.genereg.net)[16] at 90% cut-off, and those in the -0.6 kb to -0.3 kb region of the *Fthl17* promoter were analyzed by ConSite (http://consite.genereg.net)[17] at 85% cut-off.

### Statistical analysis

Statistical analysis of DNA methylation levels was performed by using the Mann- Whitney U-test. Statistical analysis of Real-time PCR and luc assay were performed by using Student’s t-Test. P-value <0.05 was considered statistically significant.

## Results

### DNA hypo-methylation of the *Fthl17* promoter induced *Fthl17* gene expression in cancer cells

**Fig 1:**
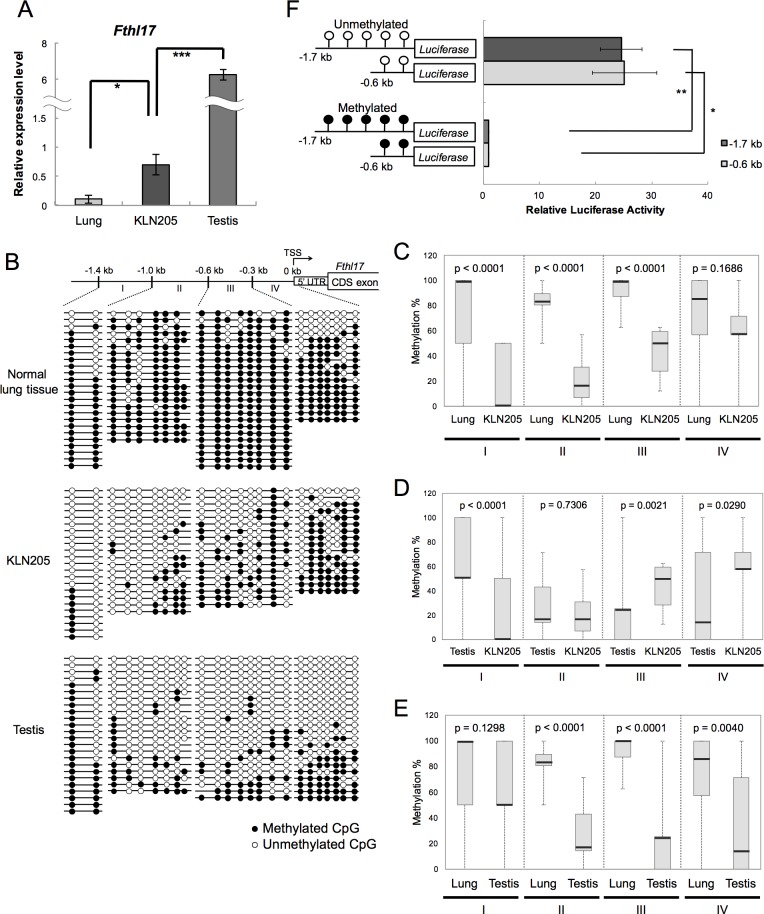
*Fthl17* gene expression was induced by promoter hypo-methylation in KLN205 cells. (A) Gene expression levels of *Fthl17* in normal lung tissue, KLN205 lung cancer cell line and testis, which were investigated by quantitative RT-PCR analysis. The primer sequences are presented in Table 1. The expression level in one sample of KLN205 cells was set as 1.0. The data were obtained from three independent experiments. *p < 0.05, ***p < 0.001. Error bars represent SEM. (B) DNA methylation status of the 5’-upstream region of *Fthl17* in normal lung tissue, KLN205 lung cancer cell line and testis, which was investigated by bisulfite sequencing analysis. The sequencing primers are presented in Table 1. Methylated and unmethylated CpGs are presented as closed circles and open circles, respectively. The 5’-upstream region of *Fthl17* is schematically shown at the top of the panel. TSS represents the transcription start site. The sequence data of each cell type were obtained from two independent experiments. (C-E) Quantitative evaluation of DNA methylation levels of the 5’-upstream region of *Fthl17* in normal lung tissues and KLN205 cells (C), testis and KLN205 cells (D), and normal lung tissues and testis (E). The region I, II, III and IV showed -1.4 kb to -1.0 kb, -1.0 kb to -0.6 kb, -0.6 kb to -0.3 kb and -0.3 kb to 0 kb, respectively. The central bars indicate medians, lower and upper limits of the boxes mark the 25^th^ and 75^th^ percentiles. The whiskers extend to the most extreme data point. Significant difference of methylation levels in each region was statistically evaluated by using the QUMA program and Mann-Whitney U-test. (F) The luciferase activity of the reporter vectors with either methylated or unmethylated *Fthl17* 5’-upstream regions (-1.7 kb or -0.6 kb) in KLN205 cells. Luciferase activity was normalized to the activity of a co-transfected Renilla phRL-TK vector. Luciferase activity from the methylated vectors was set as 1.0. The data were obtained from three independent experiments. *p < 0.05, **p < 0.01. Error bars represent SEM.

To investigate *Fthl17* expression in normal tissues, including lung and testis, and in a murine lung cancer cell line (KLN205), *Fthl17* mRNA expression levels were examined by quantitative RT-PCR. We found that *Fthl17* expression in KLN205 cells and in testis is higher than in normal lung tissues (Fig 1A). These results confirmed that mouse *Fthl17* has a characteristic CTA gene expression profile.

Because *FTHL17* was induced in human cancer cells after 5-aza-CdR treatment [9], we hypothesized that DNA methylation could regulate *Fthl17* gene expression. To examine the possible involvement of DNA methylation in the 5’-upstream region of the *Fthl17* gene, bisulfite-sequencing analysis was performed on DNA from normal murine testis and lung tissues, and KLN205 cells (Fig 1B–1E). We found that the region between -1.4 kb and -0.3 kb (the regions I, II, III) upstream from the transcription start site (TSS) was significantly more hypo-methylated in KLN205 cells than in normal lung tissues (Fig 1C). In contrast, DNA methylation of a CpG island that is located within the *Fthl17* gene body was not significantly different (S1 Fig). Therefore, the results suggest that hypo-methylation of -1.4 kb to -0.3 kb contributes to the regulation of *Fthl17* expression in KLN205 cells. In testis, the region between -0.6 kb and 0 kb (the regions III, IV) was significantly further hypo-methylated than in KLN205 cells (Fig 1D). Comparing testis with normal lung tissues, the region between -1.0 and 0 kb (the regions II, III, IV) was significantly more hypo-methylated in testis than in normal lung tissues (Fig 1E). The results suggest that hypo-methylation of -0.6 kb to 0 kb may play a role on further up-regulated expression of *Fthl17* in testis.

To verify the involvement of DNA methylation in the differentially methylated region in the regulation of *Fthl17* gene transcription, luciferase (luc) reporter assays were performed using KLN205 cells. A luc reporter construct spanning -1.7 kb (-1.7 kb to 0 kb) or -0.6 kb (-0.6 kb to 0 kb) of the *Fthl17* promoter region, with or without DNA methylation, was transfected into KLN205 cells. Both methylated luc reporter constructs showed significantly lower luc activity than the two without methylation (Fig 1F). There were no differences in luc activity between the -1.7 kb and the -0.6 kb constructs with or without DNA methylation. Thus, the results indicate that DNA hypo-methylation of the -0.6 kb to 0 kb region plays a role in induction of *Fthl17* gene expression in the cancer cells. However, it is likely that DNA hypo-methylation spanning -0.6 kb to -0.3 kb may mainly regulate *Fthl17* expression because the -0.3 kb to 0 kb region was hyper-methylated in KLN205 cells.

### DNA hypo-methylation of the *Fthl17* promoter induced *Fthl17* gene expression in germline stem cells (GSCs)

**Fig 2:**
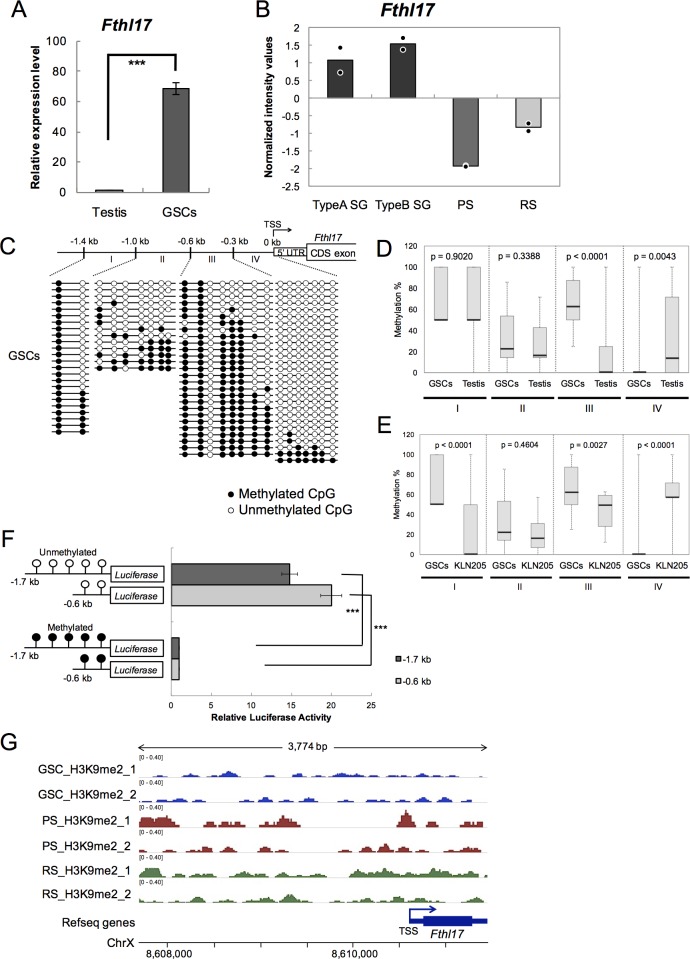
*Fthl17* gene expression was induced by promoter hypo-methylation in GSCs. (A) Gene expression levels of *Fthl17* in testis and GSCs were investigated by quantitative RT-PCR analysis. The primer sequences are presented in Table 1. The expression level in testis was set as 1.0. The data were obtained from three independent experiments. ***p < 0.001. Error bars represent SEM. (B) Normalized intensity values of *Fthl17* expression in type A spermatogonia (TypeA SG), type B spermatogonia (TypeB SG), pachytene spermatocyte (PS) and round spermatids (RS). The data obtained from published microarray data (GSE4193) were normalized by using global median scaling, and were re-analyzed using GeneSpring (Agilent). Plots show average values from two independent data sets. (C) The DNA methylation status of the 5’-upstream region of *Fthl17* in GSCs was investigated by bisulfite sequencing analysis. The sequencing primers are presented in Table 1. Methylated and unmethylated CpGs are presented as closed circles and open circles, respectively. The 5’-upstream region of *Fthl17* is schematically shown at the top of the panel. TSS represents the transcription start site. Sequence data were obtained from two independent experiments. (D-E) Quantitative evaluation of DNA methylation levels of the 5’-upstream region of *Fthl17* in GSCs and testis (D), and GSCs and KLN205 cells (E). The region I, II, III and IV showed -1.4 kb to -1.0 kb, -1.0 kb to -0.6 kb, -0.6 kb to -0.3 kb and -0.3 kb to 0 kb, respectively. The central bars indicate medians, lower and upper limits of the boxes mark the 25^th^ and 75^th^ percentiles. The whiskers extend to the most extreme data point. Significant difference of methylation levels in each region was statistically evaluated by using the QUMA program and Mann-Whitney U-test. (F) The luciferase activity of the reporter vectors with either methylated or unmethylated *Fthl17* 5’-upstream regions (-1.7 kb or -0.6 kb) in GSCs. Luciferase activity was normalized to the activity of a co-transfected Renilla phRL-TK vector. Luciferase activity from the methylated vectors was set as 1.0. The data were obtained from three independent experiments. ***p < 0.001. (G) Representative images of H3K9me2 ChIP-seq read density at the *Fthl17* promoter in GSCs, (GS, blue), pachytene spermatocyte (PS, red) and round spermatids (RS, green). ChIP-seq data (GSE69946) were re-analyzed using the Integrative Genomics Viewer (IGV).

To analyze regulation of *Fthl17* gene expression in germ cells, we performed DNA methylation analysis using cultured GSCs. The *Fthl17* expression level in GSCs was examined by quantitative RT-PCR, and was significantly higher than in testis (Fig 2A), in agreement with a previously report [5]. It suggests that the *Fthl17* gene is highly expressed in undifferentiated spermatogonial stem cells compared with differentiating spermatogenic cells. Consistent with this idea, re-analysis of published microarray data (GenBank accession number GSE4193) [18] showed that *Fthl17* gene expression is higher in spermatogonia than in pachytene spermatocytes and round spermatids (Fig 2B).

We next examined DNA methylation of the *Fthl17* promoter in GSCs (Fig 2C–2E). The region between -0.3 kb and 0 kb (the region IV) was significantly more hypo-methylated in GSCs than in testis (Fig 2D), though the region between -0.6 kb and -0.3 kb (the region III) was significantly more hyper-methylated in GSCs. Comparing with KLN205 cells, the region between -0.3 kb and 0 kb (the region IV) was also significantly more hypo-methylated in GSCs (Fig 2E), though the region I, III were significantly more hyper-methylated in GSCs. Thus, hypo-methylation of the -0.3 kb to 0 kb region is correlated with the highest expression levels of *Fthl17* in GSCs compared with testis and KLN205 cancer cells.

To verify the involvement of DNA methylation in the differentially methylated region in the regulation of *Fthl17* gene expression in GSCs, luc reporter assays were performed using GSCs. The -1.7 kb and the -0.6 kb luc reporter constructs with DNA methylation showed significantly lower luc activity than those without methylation (Fig 2F), and there were no differences in luc activity between the -1.7 kb and the -0.6 kb constructs with or without DNA methylation. Therefore, the results indicate that DNA hypo-methylation of the -0.6 kb to 0 kb region plays a role in induction of *Fthl17* gene expression in GSCs, as was seen with KLN205 cells. Because the -0.3 kb to 0 kb region was more hypo-methylated in GSCs than in KLN205 cells (Fig 2E), it is likely that the -0.3 kb to 0 kb region may be linked to the relatively high expression of *Fthl17* in GSCs.

We found that the expression of *Fthl17* in testis is markedly lower than in GSCs despite the relatively hypo-methylated state of the *Fthl17* 5’-upstream region. It is possible that *Fthl17* expression in more advanced testicular germ cells may be repressed by meiotic sex-chromosome inactivation (MSCI)-related mechanisms. To investigate this possibility, we examined MSCI-associated histone modifications within the 5’-upstream region of the *Fthl17* gene in testicular germ cells. We re-analyzed existing chromatin immunoprecipitation sequencing (ChIP-seq) data (GenBank accession number GSE69946) [19] of GSCs, pachytene spermatocytes, and round spermatids, and found that H3K9me2 in the 5’-upstream region of the *Fthl17* gene in pachytene spermatocytes and in round spermatids was indeed higher than in GSCs (Fig 2G). Meanwhile, H4K8ac (histone H4 Lysine 8 acetylation) marks and H3K27ac (histone H3 Lysine 27 acetylation) marks in the advanced testicular germ cells were similar to those in GSCs (S2 Fig). These results implicate H3K9me2 in the repression of *Fthl17* expression in advanced testicular germ cells.

### 5-Azacytidine induces an increase in *Fthl17* gene expression in cancer cells

**Fig 3:**
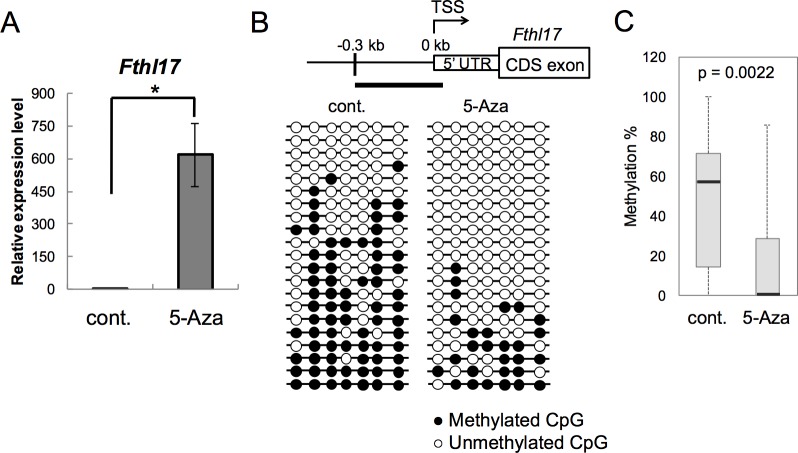
5-Azacytidine induced hypo-methylation and up-regulation of *Fthl17* gene expression in KLN205 cells. (A) Gene expression levels of *Fthl17* in KLN205 cells cultured with or without 5-Azacytidine (5-Aza) were investigated by quantitative RT-PCR analysis. The primer sequences are presented in Table 1. The expression level in one control sample (without 5-Aza) was set as 1.0. The data were obtained from three independent experiments. *p < 0.05. Error bars represent SEM. (B) DNA methylation status of the -0.3 kb to 0 kb region of the *Fthl17* promoter in KLN205 cells cultured with or without 5-Aza was investigated by bisulfite sequencing analysis. Methylated and unmethylated CpGs are presented as closed circles and open circles, respectively. The 5’-upstream region of *Fthl17* is schematically shown at the top of the panel. TSS represents the transcription start site. Sequence data were obtained from two independent experiments. (C) Quantitative evaluation of DNA methylation levels of the -0.3 kb to 0 kb region of *Fthl17* in control sample and with 5-Aza sample. The central bars indicate medians, lower and upper limits of the boxes mark the 25^th^ and 75^th^ percentiles. The whiskers extend to the most extreme data point. Significant difference of methylation levels in each region was statistically evaluated by using the QUMA program and Mann-Whitney U-test.

We next examined the effects of 5-Azacytidine (5-Aza) on DNA methylation and the expression of *Fthl17* in KLN205 cells. As shown in Fig 3A–3C, the -0.3 kb to 0 kb region became hypo-methylated in the presence of 5-Aza, and *Fthl17* mRNA levels increased significantly. The DNA methylation pattern and the expression level in the 5-Aza treated KLN205 cells were similar to those in GSCs. Therefore, these results suggest that DNA hypo-methylation of the -0.3 kb to 0 kb region of the *Fthl17* gene may result in its up-regulation in KLN205 cells.

### Prediction of possible transcription factors involved in *Fthl17* gene expression

**Fig 4:**
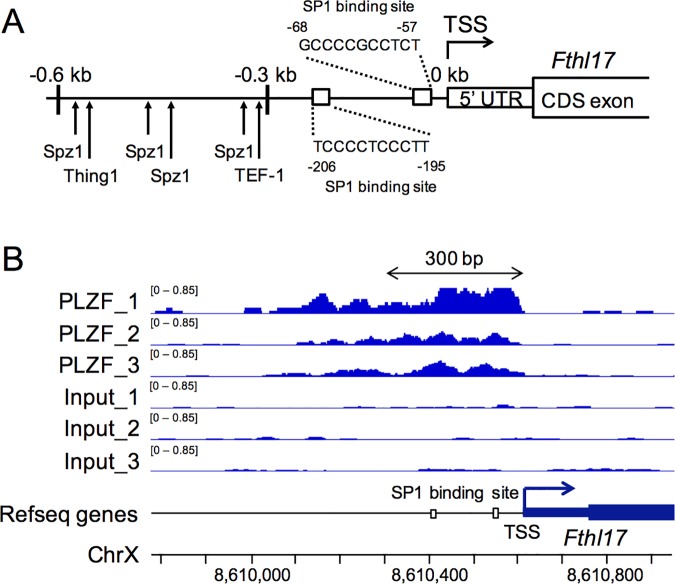
Binding sites of transcription factors in the 5’-upstream region of *Fthl17*. (A) The -0.6 kb to -0.3 kb region and the -0.3 kb to 0 kb region were analyzed by ConSite and JASPAR, respectively, to identify binding sites of transcription factors. (B) Representative images of ChIP-seq read density for PLZF in the 5’-upstream region of *Fthl17* in undifferentiated spermatogonia. ChIP-seq data (GSE73390) were re-analyzed by using the Integrative Genomics Viewer (IGV).

The results of the DNA methylation analysis and those of the luc reporter assay suggest that some transcription factors are recruited to the hypo-methylated regions of the *Fthl17*. In the case of KLN205 cells, hypo-methylation of the -0.6 kb to -0.3 kb region is important for the expression of *Fthl17*, and we identified binding sites of Spz1, Thing1 and TEF-1 in this region by using ConSite (Fig 4A). Meanwhile, hypo-methyaltion of the -0.3 kb to 0 kb region is critical for high expression of *Fthl17* in GSCs, and we found SP1 binding sites in this region by using JASPAR. Because PLZF is a transcription factor that may bind to SP1 binding site [20] and is expressed in spermatogonia [21], we re-analyzed existing chromatin immunoprecipitation sequencing (ChIP-seq) data (GenBank accession number GSE73390)[22] of undifferentiated spermatogonia, and found that PLZF was enriched in the 5’ upstream region of *Fthl17* covering the -0.3 kb to 0 kb region (Fig 4B).

## Discussion

Our results show that expression levels of the CTA gene, *Fthl17*, in lung cancer cells and in GSCs depend on methylation of the *Fthl17* TSS-proximal region. The expression of *Fthl17* in GSCs was significantly higher than that in a lung cancer cell line, KLN205 (Figs 1A and 2A). The region between -0.3 kb to 0 kb (the region IV) was more hypo-methylated in GSCs as compared to that in KLN205 cells, while the -0.6 kb to -0.3 kb region (the region III) was more hypo-methylated in KLN205 cells than that in GSCs (Figs 1B, 2C and 2D). In addition, luc reporter assay showed the hypo-methylation of the -0.6 kb to 0 kb region (the region III and IV) induced *Fthl17* up-regulation both in GSCs and KLN205 cells (Figs 1E and 2F). Thus, the results suggest that hypo-methylation of the -0.3 kb to 0 kb region results in remarkable increase of *Fthl17* expression in GSCs. Meanwhile, hypo-methylation of the -0.6 kb to -0.3 kb region may result in relatively small up-regulation in KLN205 cells. The -1.4 kb to -1.0 kb region (the region I) in KLN205 cells was significantly hypo-methylated compared with that in lung (Fig 1C), testis (Fig 1D) or GSCs (Fig 2E). However, the luc reporter assay showed that hypo-methylation in the -0.6 kb to 0 kb region (the region III and IV) was sufficient for up-regulated expression of *Fthl17* gene in KLN205 cells. In addition, methylation status in the region I did not correlate to its expression levels in GSCs and KLN205 cells. Thus, hypo-methylation in the region I is not involved in its up-regulation in KLN205 cells. Taken together, the results suggest that hypo-methylation of the different 5’-upstream regions of *Fthl17* is involved in its differential expression levels, and hypo-methylation of the TSS-proximal region (-0.3 kb to 0 kb) may be critical for its high level expression. This idea was further supported by the data that induced hypo-methylation of the TSS-proximal region by 5-Azacytidine (5-Aza) up-regulated *Fthl17* gene expression in KLN205 cells (Fig 3).

5-Aza induces global hypo-methylation, and therefore it is possible that hypo-methylation of a putative *Fthl17* distal enhancer, and/or up-regulation of genes that encode transcription factors that regulate *Fthl17*, may also be involved in up-regulation of *Fthl17* expression in response to 5-Aza. Indeed, different transcription factors may bind to the -0.6 kb to -0.3 kb or the -0.3 kb to 0 kb region in a DNA methylation-dependent manner in KLN205 cells and in GSCs to control *Fthl17* expression levels.

In the lung cancer cells, abnormal hypo-methylation of the *Fthl17*–0.6 kb to -0.3 kb region may be due to insufficient recruitment of DNA methyltransferases and/or their co-cofactors to this region, which may cause up-regulation of *Fthl17* expression.

Testis contain several different types of cells, including spermatogonia, spermatocytes, spermatids, sertoli cells, and Leydig cells. Because the expression of *Fthl17* in GSCs is much higher than in testis, *Fthl17* is likely preferentially expressed in spermatogonial stem cells, and microarray data supported this idea (Fig 2B). Although the DNA methylation level of the -0.3 kb to 0 kb region of the *Fthl17* gene in GSCs was lower than that in testis, the difference cannot be explained by the number of spermatogonia in testicular cells, because only about 2.1% of testicular cells are spermatogonia [23,24], and thus *Fthl17* may also be hypo-methylated to some extent in differentiating testicular cells. Therefore, additional repressive epigenetic control may play a role in repression of *Fthl17* expression in testicular cells other than spermatogonia.

Because *Fthl17* is an X-linked gene, it may be repressed via formation of an XY-body in which H3K9me2 is enriched, associated with meiotic sex-chromosome inactivation (MSCI) in spermatocytes from late-pachytene stage [18]. Most X-linked genes are silenced from the pachytene stage of meiosis to post-meiosis, and it is considered that these genes may be regulated by histone modifications, such as deacetylation of histone H3 and H4 [25], H3K9me2 [25,26] and H2A ubiquitination [27,28] during MSCI. In agreement with this, H3K9me2 enrichment at the TSSs of X chromosome genes in GSCs is comparable to that of autosomal genes, but H3K9me2 was more enriched on X chromosome genes compared with autosomal genes in pachytene spermatocytes and in round spermatids [19]. While reductions of H4K8ac and H3K27ac at the *Fthl17* 5’-upstream region were not detected (S2 Fig), enrichment of H3K9me2 within the *Fthl17* 5’-upstream region in pachytene spermatocytes and round spermatids (Fig 2G) was consistent with the above-mentioned observation. Therefore, *Fthl17* expression might be repressed by H3K9me2 modification in spermatocytes and spermatids despite of hypo-methylation of the -0.3 kb to 0 kb region.

We propose that hyper-methylation of the -0.6 kb to 0 kb region is crucial for repression of *Fthl17* expression in normal somatic tissues. In addition, *Fthl17* expression could also be repressed by histone modifications, including H3K9me2. However, the possibility of low level expression of critical transcriptional regulators of *Ftlh17* expression in normal somatic cells and differentiating spermatogenic cells cannot be excluded. *In silico* analysis indicated that there were binding sites of critical transcription factors in the hypo-methylated regions of *Fthl17* in KLN205 cells and GSCs. Among them, Spz1 whose binding sites were found in the –0.6 kb to -0.3 kb region (Fig 4A), is expressed in several murine cancer cell lines and stimulates cell proliferation and tumorigenesis [29]. Meanwhile, Plzf which likely recognizes SP1 binding sites in the -0.3 kb to 0 kb region (Fig 4A and 4B), is a sprermatogonia specific transcription factor and regulates self-renewal of spermatogonia [21]. Although functions of *Fthl17* gene are so far unknown, the results suggest that *Fthl17* is involved in cell proliferation through regulation by Spz1 and Plzf in cancer cells and in GSCs, respectively. The regulatory mechanisms controlling DNA methylation levels within the *Fthl17* 5’-upstream region, and functions and recruitment of the above-mentioned predicted transcription factors on the regulatory regions of *Fthl17* in GSCs and in cancer cells, remain to be clarified in future experiments.

## Supporting information

S1 Fig DNA methylation status of *Fthl17* CpG islands in normal lung tissues and KLN205 cells. (A) Methylated and unmethylated CpGs are presented as closed circles and open circles, respectively. The region of the *Fthl17* gene body including CpG islands is schematically shown at the top of the panel. TSS represents the transcription start site. Sequence data were obtained from a single sample. (B) Quantitative evaluation of DNA methylation levels of the 0.1 kb to 0.4 kb region of *Fthl17* in normal lung tissues and KLN205 cells. The central bars indicate medians, lower and upper limits of the boxes mark the 25^th^ and 75^th^ percentiles. The whiskers extend to the most extreme data point. Significant difference of methylation levels in each region was statistically evaluated by using the QUMA program and Mann-Whitney U-test.
(TIF)

S2 Fig Histone acetylation status of the *Fthl17* promoter in spermatogenic cells. (A) Representative images of H4K9ac ChIP-seq read density at the *Fthl17* promoter in GSCs, (GS, blue), pachytene spermatocyte (PS, red) and round spermatids (RS, green). ChIP-seq data (GSE69946) were re-analyzed using the Integrative Genomics Viewer (IGV). (B) Representative images of H3K27ac ChIP-seq read density at the *Fthl17* promoter in spermatogonia, (SG, blue), spermatocytes (SC, red) and spermatids (ST, green). ChIP-seq data (GSE49621) were re-analyzed using the Integrative Genomics Viewer (IGV).
(TIF)
