# Supplementary figures and images for: DNA methylation of the Fthl17 5’-upstream region regulates differential Fthl17 expression in lung cancer cells and germline stem cells
Source: PLoS One. 2017 Feb 16;12(2):e0172219. doi: 10.1371/journal.pone.0172219 (PMC5312872; doi:10.1371/journal.pone.0172219)

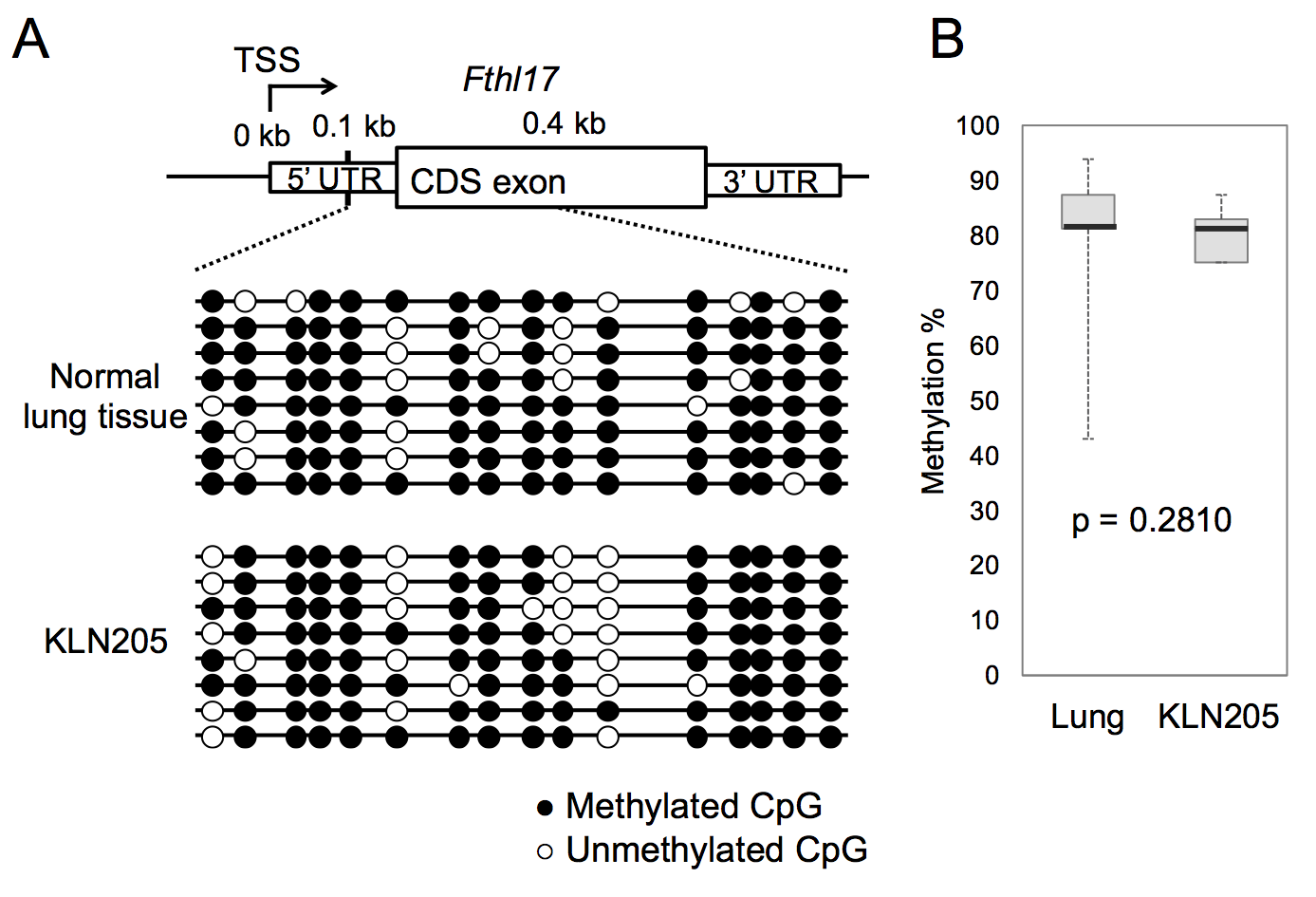

Supplement: S1 Fig — (A) Methylated and unmethylated CpGs are presented as closed circles and open circles, respectively. The region of the Fthl17 gene body including CpG islands is schematically shown at the top of the panel. TSS represents the transcription start site. Sequence data were obtained from a single sample. (B) Quantitative evaluation of DNA methylation levels of the 0.1 kb to 0.4 kb region of Fthl17 in normal lung tissues and KLN205 cells. The central bars indicate medians, lower and upper limits of the boxes mark the 25th and 75th percentiles. The whiskers extend to the most extreme data point. Significant difference of methylation levels in each region was statistically evaluated by using the QUMA program and Mann-Whitney U-test. (TIF) [file pone.0172219.s001.tif]

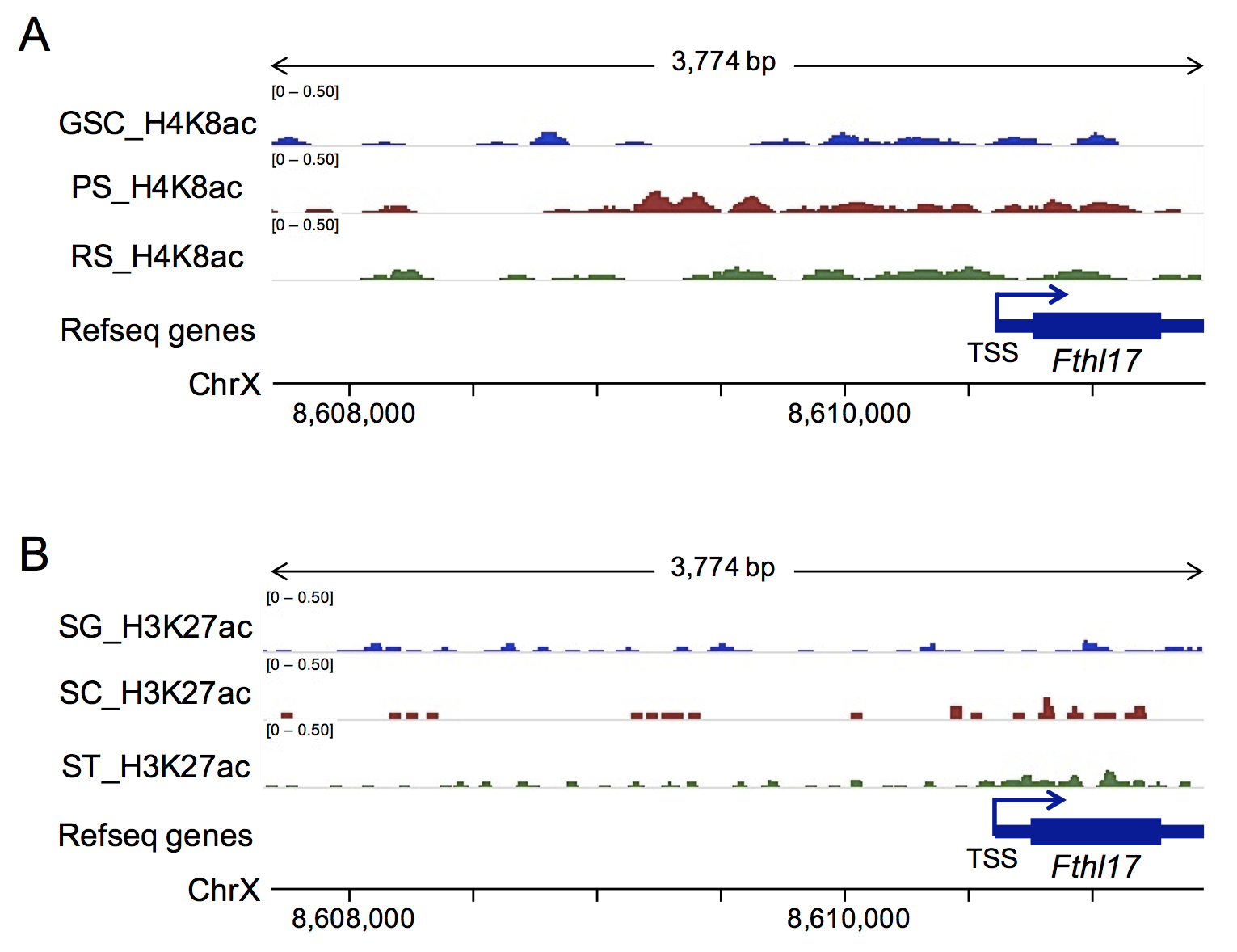

Supplement: S2 Fig — (A) Representative images of H4K9ac ChIP-seq read density at the Fthl17 promoter in GSCs, (GS, blue), pachytene spermatocyte (PS, red) and round spermatids (RS, green). ChIP-seq data (GSE69946) were re-analyzed using the Integrative Genomics Viewer (IGV). (B) Representative images of H3K27ac ChIP-seq read density at the Fthl17 promoter in spermatogonia, (SG, blue), spermatocytes (SC, red) and spermatids (ST, green). ChIP-seq data (GSE49621) were re-analyzed using the Integrative Genomics Viewer (IGV). (TIF) [file pone.0172219.s002.tif]
